# Can new consumption promote urban industrial resilience? Empirical evidence from pilot cities of information consumption

**DOI:** 10.1371/journal.pone.0323101

**Published:** 2025-05-28

**Authors:** Chao Han, Hang Su

**Affiliations:** 1 School of Economics, Shandong University of Finance and Economics, Jinan, China; 2 School of Management, Guizhou University of Commerce, Guiyang, China; Zhejiang A&F University, CHINA

## Abstract

The rapid advancement of digital technology and its widespread application have led to digitalization, personalization, and customization in the demand side of China’s economy. Enhancing industrial resilience through new types of consumption is of great practical significance for expanding domestic demand and promoting high-quality, sustainable economic growth in China. This study examines the impact of the Information Consumption Pilot City (ICPC) policy as a quasi-natural experiment on urban industrial resilience, employing the difference-in-difference (DID) method for empirical analysis. The findings reveal that the ICPC policy significantly enhances the level of urban industrial resilience. Heterogeneity tests indicate that this enhancement effect is particularly pronounced in eastern, central, and larger urban regions. Furthermore, the ICPC policy primarily strengthens urban industrial resilience through three mechanisms: information development, entrepreneurial agglomeration, and digital financial effects. This study contributes to the literature on new consumption and urban industrial resilience in the digital economy, evaluates the economic impacts of pilot policies on information consumption, and offers valuable implications for policymakers.

## 1. Introduction

The resilience of urban industries has emerged as a critical issue in modern urban economic governance. In recent years, the deepening of globalization and the increasing complexity of geopolitical dynamics have exposed urban industrial chains to unprecedented challenges, such as supply chain disruptions, rising cross-border trade barriers, and heightened market demand uncertainties. Additionally, the frequent occurrence of public health crises and natural disasters underscores the pressing need to enhance industrial resilience [1]. In this context, strengthening industrial resilience is not only essential for the sustainable development of individual enterprises but also crucial for maintaining urban economic stability and societal well-being [2]. However, traditional industrial management models often prove inadequate in addressing these emerging challenges [3]. The rapid advancement of digital technologies, particularly their increasing breadth and depth of application, holds significant potential for optimizing resource allocation across industries, thereby enhancing the resilience and security of regional industries.

Information consumption, as a vital component of emerging consumption patterns, has become a key driver of economic growth and a catalyst for industrial transformation and upgrading [4]. In 2013, the State Council of China (SCC) issued the *Several Opinions on Promoting Information Consumption to Expand Domestic Demand*, marking the first time that a national strategy explicitly advocated for the vigorous development of information consumption. This initiative aimed to foster the widespread adoption and deepening of information consumption across regions through pilot programs, to optimize economic structures and advance industrial upgrading [5]. In light of rapid digital advancements, information consumption—anchored in platforms like internet devices—leverages emerging digital technologies such as cloud computing and the Internet of Things (IoT). These technologies not only promote high-quality development within the information industry but also enhance the digitization of each link in the industrial chain. This, in turn, optimizes resource allocation by leveraging inter-industry linkages and strengthens the interconnectedness of industrial chains, thereby enhancing the resilience of urban economies against external shocks [6,7]. In the context of China’s insufficient domestic demand and heightened policy uncertainty, investigating the impact of information consumption on urban industrial resilience is of significant practical relevance.

Industrial resilience is fundamentally a form of adaptive resilience, manifested in an industry’s capacity to withstand, recover from, and adjust to external shocks [8,9]. Most scholars define industrial resilience in terms of the industry’s resistance to shocks and its recovery capacity afterward [10,11]. While there is no consensus on the measurement of industrial resilience, many studies adopt a comprehensive evaluation approach [12,13]. Industrial resilience plays a crucial role in ensuring urban economic stability, though its enhancement is influenced by a serious of factors [14]. Structurally, factors such as industrial diversity, node redundancy, and the geographic breadth of supply chains are key to improving resilience. For instance, Sheffi and Rice [15] argue that a diversified supply chain structure can effectively mitigate risks, thereby enhancing industrial resilience. In terms of socio-economic factors, research has explored the influence of new infrastructure [16,17] and population agglomeration [18] on the resilience of urban industrial chains. From a policy and governance perspective, studies primarily examine the role of property rights systems [19] and free trade zones [20] in strengthening urban industrial resilience. However, limited attention has been paid to the role of information consumption in enhancing industrial resilience.

While the existing literature provides a solid theoretical foundation for industrial resilience research, it remains insufficient in exploring how information consumption can enhance industrial resilience. To address this gap, this study utilizes data from China’s prefecture-level cities for the period 2011–2022, employing the ICPC policy as a quasi-natural experiment to empirically examine its impact on industrial resilience. Using the difference-in-differences (DID) method, the study also aims to uncover the underlying mechanisms through which information consumption influences resilience. Additionally, it investigates how new forms of consumption contribute to urban industrial resilience through three key channels: information development, entrepreneurial agglomeration, and digital finance. Lastly, considering the differences in urban geographic location and resource endowment, we further examine the heterogeneity of new consumption affecting urban industrial resilience.

The potential marginal contributions of this study are as follows: First, while existing research predominantly examines the drivers of urban industrial resilience from a supply-side perspective [21,22], limited attention has been paid to this issue from the demand side. This paper innovatively approaches the topic by incorporating information consumption, a new form of demand in the digital era, into the analytical framework of urban industrial resilience. The empirical findings based on this framework demonstrate that information consumption significantly enhances urban industrial resilience. This not only enriches the literature by adding a demand-side perspective to the factors influencing industrial resilience but also extends the empirical evidence linking policy and governance factors to high-quality industrial development. Second, prior studies primarily focus on the effects of information consumption on innovation and entrepreneurship [23,24], industrial diversification [25], industrial structure optimization [26,27], high-quality development of the economy [28,29] and green development [30,31], with insufficient attention paid to its impact on urban industrial resilience. By empirically investigating the effect of the ICPC policy on industrial resilience from the perspective of sustainable urban development, this study fills a critical gap and enriches the literature on the role of information consumption in promoting urban resilience. Finally, this study explores the mechanisms through which information consumption enhances urban industrial resilience, focusing on the roles of information development, entrepreneurial agglomeration, and digital finance. It further analyzes the heterogeneous effects of information consumption on industrial resilience across different cities. By opening new avenues for understanding how information consumption fosters industrial resilience, this research offers valuable insights for promoting sustainable urban development empowered by digital consumption. Additionally, it provides a theoretical foundation for expanding ICPC pilots and advancing the digital transformation of consumption.

## 2. Theoretical analysis and research assumptions

### 2.1. Policy background

With the rapid advancement of information technology, information consumption has emerged as a new driving force for stimulating domestic demand and promoting economic growth. To accelerate informatization and deepen the application of information technology in the economy, the Chinese government has introduced a series of policies to promote information consumption. Among these, the ICPC policy stands out as a key initiative, aimed at exploring new avenues for leveraging information consumption to drive economic development through early-stage pilots. In 2013, the SCC issued the *Several Opinions on Promoting Information Consumption and Expanding Domestic Demand*, explicitly advocating for information consumption as a critical pathway to accelerate the integration of informatization with industrialization and to promote the establishment of ICPC. Subsequently, the Ministry of Industry and Information Technology (MIIT) approved 68 cities as the first batch of ICPC pilot cities in 2013, followed by an additional 36 cities in the second batch in 2015. The successful implementation of the ICPC policy has also been facilitated by strong support and active participation from local governments, which have customized policies to align with their economic foundations and industrial characteristics. These local initiatives, including increased financial investment, optimized business environments, and strengthened information infrastructure, have contributed to the rapid growth of the information consumption industry.

The ICPC policy is also rooted in the requirements of China’s current stage of economic development. As China’s economy enters a “new normal,” the traditional growth model faces challenges, and there is an urgent need to achieve sustainable development through the expansion of domestic demand and economic restructuring. Information consumption, as an emerging consumption hotspot, not only meets the increasing consumption needs of residents but also stimulates the development of related industries, forming new growth drivers for the economy. Thus, selecting cities as pilots to foster the growth of the information consumption market through targeted policy support has become a key initiative in China’s strategy for economic transformation. These pilot cities have experienced rapid growth in the information consumption market, alongside significant improvements in the concentration and innovation capacity of the information industry, providing valuable insights and models for other cities to follow.

### 2.2. Research hypotheses

#### 2.2.1. ICPC and industrial resilience.

As a key initiative for promoting the digital transformation of cities, the ICPC policy enhances industrial resilience through three main channels. First, it broadens the application and deepens the penetration of information technology in pilot cities, thereby accelerating the digital transformation and intelligent upgrading of industrial chains. This transformation strengthens the industrial chain’s resilience by improving information accessibility and flow, making it less vulnerable to external shocks [32,33]. Second, the ICPC policy, as a critical driver of new consumption and a lever for economic circulation, promotes the upgrading of consumption structures and the expansion of domestic demand by stimulating intelligent, personalized, and high-end consumption. This, in turn, drives the extension and development of industries [24,34].

Moreover, from the perspective of resource agglomeration and informatization, the ICPC policy accelerates the development of information infrastructure in pilot cities and fosters the integration of information technology across various industrial chain links. This facilitates the agglomeration and sharing of innovative resources, while providing enterprises with a more efficient external environment, thus lowering the costs of entrepreneurial activities. The resulting denser industrial chain network enhances the innovation capacity of urban industries, promoting effective collaboration and increasing adaptability to market fluctuations, ultimately improving resilience [35]. Therefore, we propose the following hypothesis:

H1: The ICPC policy can effectively enhance the urban industrial resilience.

#### 2.2.2. Mechanism hypothesis.

**Fig 1:**
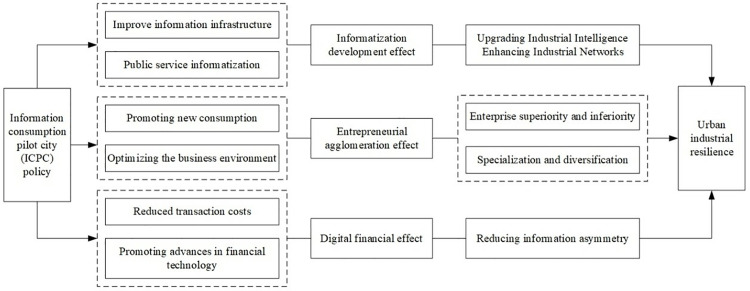
Mechanisms of the ICPC policy on urban industrial resilience.

Based on the specific objectives of the ICPC policy and the analysis outlined in the previous sections, this study seeks to explore the mechanisms through which the ICPC policy enhances industrial resilience. The analysis will focus on three key aspects: information development, entrepreneurial agglomeration, and digital finance, as illustrated in Fig 1.

(1) Information development effect. The ICPC policy explicitly outlines several targeted measures aimed at accelerating the improvement of information infrastructure and enhancing the informatization of public services in pilot cities. For instance, the policy emphasizes the construction of high-speed broadband networks, the deployment of 5G infrastructure, and the promotion of smart city platforms [30,31]. These measures not only facilitate the overall informatization development of these cities but also create a robust foundation for digital transformation across various industries. Firstly, an increase in the level of informatization facilitates the digital transformation and intelligent upgrading of industries, significantly improving the speed of information acquisition, transmission, processing, and feedback across various links of the industrial chain [23]. This enables enterprises to respond more rapidly to market changes and adjust their production strategies accordingly, thus enhancing the adaptability and flexibility of the industrial chain.

Secondly, informatization promotes efficient collaboration and optimized resource allocation among the different nodes of the industrial chain. Through data sharing and information integration, supply chain management and collaborative innovation capabilities are strengthened, reducing uncertainties and risks in the production process [36]. Particularly in the face of emergencies or external shocks, a high level of informatization enables the industrial chain to quickly mobilize and integrate resources, forming a rapid response mechanism that mitigates potential economic losses [37]. The deep application of information technology also allows enterprises to innovate production processes and optimize operational models, further enhancing the competitiveness and sustainable development capacity of the industrial chain. Therefore, we propose the following hypothesis:

H2: The ICPC policy enhances the urban industrial resilience by promoting the information development.

(2) Entrepreneurial agglomeration effect. Firstly, ICPC cities foster new digital consumption formats and, coupled with promising economic development prospects, generate a substantial number of job opportunities for the region. This attracts the agglomeration of production factors such as labor and capital, providing essential resource support for entrepreneurial activities. Secondly, The ICPC policy creates a digitalized, information-driven business environment that effectively reduces information asymmetry and lowers entrepreneurial costs, thereby stimulating the agglomeration of entrepreneurial activities in cities [24]. Specifically, these policies equip cities with efficient digital infrastructure, significantly enhancing the information mobility and resource-sharing capabilities among enterprises. This enables startups to rapidly access market information, technical support, and financial resources [5,38], accelerating the industrialization of innovative outcomes. Additionally, the expansion of information consumption continuously generates new market demands, attracting more enterprises to enter related fields.

Existing literature indicates that the agglomeration of entrepreneurial activities effectively enhances the resilience of urban industrial chains [39,40]. When startups with digital technology advantages enter the market, increased information flows and technical exchanges encourage firms to overcome inertia and strengthen collaborative innovation, promoting closer cooperation and resource-sharing among upstream and downstream participants in the industrial chain. This coordinated and efficient collaboration network mitigates industrial risks and improves the overall ability of the industrial chain to withstand market fluctuations and external shocks [41]. Furthermore, entrepreneurial agglomeration promotes diversification and specialization within the industrial chain. The refinement of the intra-industry division of labor enhances the overall efficiency of urban industries and strengthens complementarity and coordination within the industrial chain, enabling it to maintain greater adaptability and resilience in complex and volatile market conditions [40,42]. Therefore, the following hypothesis is proposed:

H3: The ICPC policy enhances the urban industrial resilience by promoting entrepreneurial agglomeration.

(3) Digital finance effect. The implementation of the ICPC policy significantly enhances the digitalization and inclusiveness of financial services. First, by promoting the integration of municipal e-government platforms with digital payment systems, the policy fosters the widespread adoption of mobile payments, contactless transactions, and intelligent financial services. Specific initiatives, such as subsidies for digital payment infrastructure and tax incentives for fintech firms, have accelerated the deployment of financial technologies in urban economies. Second, the establishment of government-led credit information sharing platforms, particularly through municipal big data centers, improves financial institutions’ ability to conduct data-driven credit assessments and risk management. This reduces information asymmetry, lowers transaction costs, and expands the accessibility of financial services. Third, regulatory policies under the ICPC framework encourage collaboration between traditional financial institutions and fintech enterprises, facilitating innovation in digital lending, supply chain finance, and inclusive credit products.

With the expansion of digital financial infrastructure, enterprises, particularly small and medium-sized enterprises (SMEs), can access financing and other financial services more seamlessly. On one hand, this reduction in financing costs alleviates financial strain on capital chain, thereby strengthening the resilience of industries to risks [7]. Concurrently, financial technologies, such as digital inclusive finance, utilize big data, artificial intelligence, and other digital tools to mitigate resource misallocation and effectively facilitate inter-firm collaboration and resource sharing [43]. This establishes a solid foundation for improving the coordination and adaptability of urban industries [44]. Consequently, industrial chains maintain stability and resilience amid market fluctuations and external shocks through more agile capital operations and resource allocations. On the other hand, digital finance, with its high accessibility, reduces transaction costs and enhances payment convenience, promoting the upgrading of residents’ consumption capacity and structure [45]. This consumption upgrade not only expands market demand but also spurs new demands and business formats, prompting enterprises to engage in technological innovation and product optimization. This accelerates the upgrading and renewal of products and services, driving the industrial structure towards high-end and diversified development [44]. Such optimization strengthens industries’ adaptability and recovery capacity in response to external risks, further enhancing overall resilience. Based on the above analysis, the following hypothesis is proposed:

H4: The ICPC policy can effectively enhance the urban industrial resilience by promoting the development of digital finance.

## 3. Research design

### 3.1. Model design

The primary objective of this study is to examine the impact of ICPC policy on urban industrial resilience, while also exploring its underlying mechanisms and heterogeneity. Building upon previous research by Brown and Greenbaum [40] and Li [46], we construct the following econometric model to empirically test this primary objective:


$${Res}_{it}=\alpha_{0}+\alpha_{1}{Did\_inf}_{it}+\beta {Controls}_{it}+\lambda_{t}+\mu_{i}+\varepsilon_{it}$$ (1)

Where i represents the city, and t denotes the year. ${Res}_{it}\;$is the dependent variable, indicating the level of industrial resilience of city i in year t. ${Did\_inf}_{it\;}\;$serves as the explanatory variable. Controls represents a series of control variables that may bias the baseline results. $\lambda_{t}\;$and $\mu_{i}\;$denote the year-fixed effects and city-fixed effects, respectively. $\alpha_{0}\;$is the regression constant term, and $\varepsilon_{it}\;$represents the random disturbance term.

### 3.2. Variable definition

#### 3.2.1. Explained variable.

Urban industrial resilience refers to the capacity of an industrial system to maintain stability and continuity in the face of external shocks, such as economic crises, natural disasters, or supply chain disruptions [47]. This resilience not only reflects the system’s ability to withstand risks but also encompasses its speed of recovery and adaptability to new environments post-crisis. Given the multifaceted nature of resilience, a single indicator may inadequately capture its complexity, necessitating a multidimensional comprehensive assessment. Scholars generally acknowledge that both shock resistance and transformational recovery capacity are critical dimensions of urban industry resilience [46,48]. Therefore, this study, drawing on the frameworks established by Di et al.[48] and Li [46], measures resilience comprehensively through the dimensions of shock resistance and transformational recovery capacity, utilizing available prefecture-level city-data.

Shock resistance is assessed using the city’s industrial diversity index, which is represented by the reciprocal of industrial concentration, specifically the Herfindahl-Hirschman Index. This metric evaluates the relative significance of various industries within the urban economy, reflecting the capacity for resource allocation and risk diversification in response to external shocks. Additionally, transformational recovery capacity is measured by the number of authorized invention patents—a metric widely recognized in the academic community [17,20,49]. Its theoretical foundation is primarily reflected in three aspects. First, the number of authorized invention patents reflects the development level of the industrial chain in terms of innovation resource investment, the construction of an innovative environment, and skill upgrading. Enhanced innovation accelerates the development and refinement of new products, thereby improving the efficiency of industrial renewal and iteration [46,50]. Second, patent counts indicate the diversification of technological innovation and knowledge accumulation within a region. As regional technological capabilities and reserves become increasingly diversified, the depth and breadth of technological knowledge expand, enriching the regional technological structure. This not only signifies breakthroughs in individual technologies but also demonstrates an overall improvement in regional technological levels, thereby providing a solid knowledge base for industrial entities to adjust and restore their interrelationships to a normal or even more optimal state in the face of potential market risks and uncertainties [40]. Third, high levels of patent activity are often accompanied by the emergence of new technologies and industries, which can accelerate the diversification and adjustment of the regional industrial structure. Under the pressure of external shocks, new technologies often become an important tool for industries to seek breakthroughs and rapid development [37,51]. In summary, using the number of authorized invention patents as an indicator for transformational recovery capability not only directly reflects the level of technological innovation but also reveals the underlying mechanisms by which regions construct complex technological networks and promote industrial diversification, fully embodying their capacity to transform and upgrade in response to external environmental changes.

Finally, following the approach of Yang and Liu [17], this study employs the panel entropy weight method to comprehensively calculate the comprehensive index of *Res* (where, the weights of shock resistance and transformational recovery capacity were weighted 0.159 and 0.841, respectively). The calculation steps are as follows:

First, data standardization:


$$Positive\;Indicator:{\;X}_{ijt}^{\prime }=\frac{X_{ijt}-min(X_{1j},X_{2j},\;\ldots \ldots ,\;X_{nj})}{\max\left(X_{1j},X_{2j},\;\ldots \ldots ,\;X_{nj}\right)-min(X_{1j},X_{2j},\;\ldots \ldots ,\;X_{nj})}$$ (2)

$$Negative\;Indicator:\;X_{ijt}^{\prime }=\frac{\max\left(X_{1j},X_{2j},\;\ldots \ldots ,\;X_{nj}\right)-X_{ijt}}{\max\left(X_{1j},X_{2j},\;\ldots \ldots ,\;X_{nj}\right)-min(X_{1j},X_{2j},\;\ldots \ldots ,\;X_{nj})}$$ (3)

Where, *X’* represents the standardized value of indicator j for city i in year t, i = 1, …, n; j= 1, …, m; t= 1, …, s.

Second, calculation of indicator proportion:


$${P_{ijt}=X}_{ijt}^{\prime }/\sum_{t}\sum_{i}X_{ijt}^{\prime }$$ (4)

Third, calculation of information entropy:


$$e_{j}=-\frac{1}{\ln(ns)}\sum_{t}\sum_{i}P_{ijt}\ln\left(P_{ijt}\right)$$ (5)

Fourth, calculation of information utility value:


$$g_{j}=1-e_{j}\;$$ (6)

Fifth, determination of indicator weights:


$$w_{j}=g_{j}/\sum_{j}g_{j\;}$$ (7)

Sixth, calculation of the comprehensive industrial resilience score:


$${RS}_{it}=\sum_{j}w_{j}X_{ijt}^{\prime }$$ (8)

#### 3.2.2. Explanatory variable.

The explanatory variable in this study is Did_inf, a dummy variable used to measure whether a city has been designated as an ICPC pilot. If a city is included in the list of ICPC pilots, Did_inf takes a value of 1 in the year it is included and for all subsequent years; otherwise, it takes a value of 0.

#### 3.2.3. Control variables.

Building on the practices of previous studies [5,30,31], to ensure the reliability of the benchmark regression results, this paper controls for a series of variables that may potentially affect the urban industrial resilience. Specifically, these include: economic development level (Pgdp), measured by the natural logarithm of the city’s per capita GDP; financial development (Finance), proxied by the ratio of total loans from banks and other financial institutions to GDP, following common practices in existing research; human capital (Edu), measured by the natural logarithm of educational expenditure; fiscal pressure (Pressure), quantified as the ratio of general government revenue to GDP; average wage (Wage), measured by the natural logarithm of the average wage.

### 3.3. Sample selection and data sources

Given the external shocks to the economic environment and the availability of urban data, this study collects panel data from 281 prefecture-level cities in China for 2011–2022 to examine the impact and underlying mechanisms of the ICPC policy on industrial resilience. The list of ICPC cities is sourced from official documents published on the website of the MIIT of China and compiled manually for accuracy. The remaining data are derived from the “China City Statistical Yearbook” and the EPS database. To address any missing values, relevant statistical yearbooks from the corresponding years are consulted. Descriptive statistics of the main variables utilized in this study are presented in Table 1.

**Table 1:** Descriptive statistics.

Variable	Obs	Mean	S. D.	Min	Max
Res	3372	0.034	0.065	0.002	0.720
Did_inf	3372	0.218	0.413	0	1
Pgdp	3372	10.811	0.732	9.219	19.447
Edu	3372	13.2	0.797	9.906	16.276
Finance	3372	1.065	0.588	0.310	4.459
Wage	3372	10.999	0.377	9.753	14.081
Pressure	3372	0.076	0.026	0.025	0.175

## 4. Empirical results

### 4.1. Baseline regression

Table 2 presents the regression results examining the impact of the ICPC policy on urban industrial resilience. The analysis is conducted in a stepwise manner, progressively incorporating control variables as well as city and year-fixed effects into the model. The estimated coefficient of the information consumption pilot (Did_inf) is significantly positive at the 1% level, indicating that the ICPC policy substantially enhances the industrial resilience of the pilot cities. Specifically, after controlling for the effects of other variables, the coefficient for Did_inf is 0.018, suggesting that the industrial resilience of the pilot cities under the ICPC policy is 0.018 higher than that of non-pilot cities. This confirms that new forms of consumption, represented by information consumption, can play a significant and positive role in promoting urban industrial resilience. This result aligns with previous studies by some scholars [52,53]. A possible reason is that, on the one hand, the pilot policies help strengthen the construction of regional digital infrastructure and promote the development of the urban digital economy, thereby driving the high-quality development of urban industries [54,55], which provides cities with strong resistance to risks. On the other hand, the ICPC cities use digital technologies to enhance the allocation of market resources, stimulate entrepreneurial activity, and improve technological innovation capabilities [7,16,21,30], significantly improving the diversity and innovation resilience of urban industries. This endows cities with robust capabilities to cope with uncertainties and enhances resilience. Therefore, the construction of information consumption pilots contributes to promoting the resilience of urban industrial chains, validating Hypothesis H1.

**Table 2:** Baseline regression results.

	(1)	(2)	(3)
	**Res**	**Res**	**Res**
Did_inf	0.048^***^	0.020^***^	0.018^***^
	(18.640)	(3.658)	(3.563)
Pgdp			-0.020^**^
			(-2.361)
Edu			0.065^***^
			(3.288)
Finance			0.011^*^
			(1.681)
Wage			-0.007
			(-1.334)
Pressure			-0.190^***^
			(-2.601)
Constant	0.024^***^	0.030^***^	-0.539^**^
	(19.971)	(25.235)	(-2.533)
City-FE	NO	YES	YES
Year-FE	NO	YES	YES
N	3372	3372	3372
R^2^	0.093	0.735	0.752

Note:

***,

**, and

*indicate significance levels of 1%, 5%, and 10% respectively. The values in parentheses are T-test values clustered at the city level. The same applies below.

### 4.2. Analysis of parallel trends and dynamic effects

To consider the ICPC policy as an effective exogenous shock, it is essential to satisfy the parallel trend assumption. This means that the trends of industrial resilience in ICPC pilot cities and non-pilot cities should exhibit similar patterns before the implementation of the policy. To test this assumption, this study follows the methodologies outlined by Jacobson et al. [56] and Beck et al. [57] by employing an event study approach. The econometric model used for this analysis is specified in Equation (9):


$${Res}_{it}=\alpha_{0}+\alpha_{k}\sum_{k=-4}^{k=8}{Did\_inf}_{it}^{k}+\beta {Controls}_{it}+\lambda_{t}+\mu_{i}+\varepsilon_{it}\;$$ (9)

**Fig 2:**
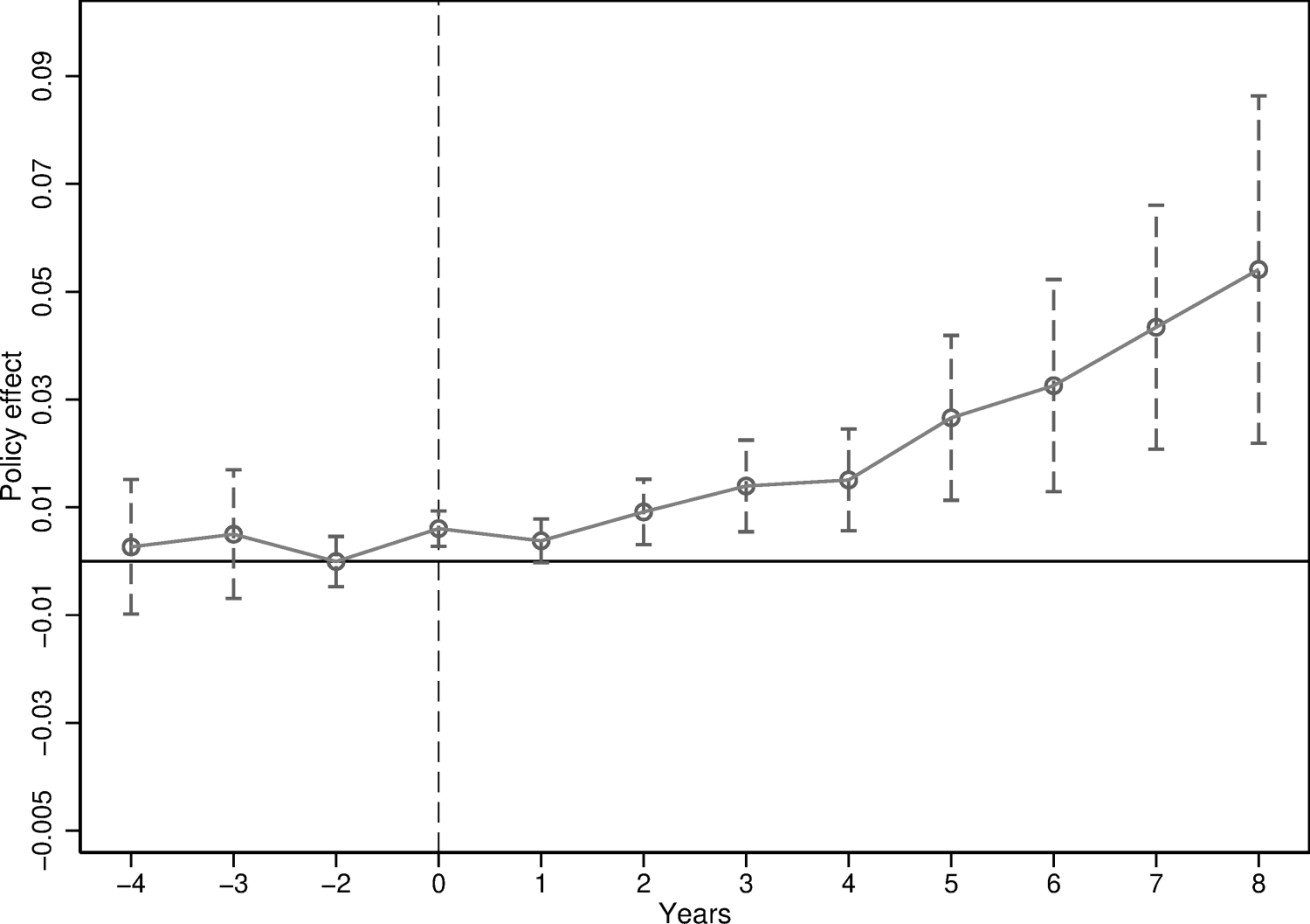
Parallel trend test.

Fig 2 presents the estimated values of the coefficient $\alpha_{k}$ along with their 95% confidence intervals. Before the implementation of the ICPC policy, the confidence intervals for all $\alpha_{k}$ estimates include 0, indicating a failure to reject the null hypothesis that it equals 0. This result suggests that there were no systematic differences in industrial resilience between pilot cities and non-pilot cities before the policy intervention, supporting the assertion that both groups shared similar trends before the introduction of the ICPC policy.

Furthermore, the confidence interval of the estimated value of $\alpha_{k}$ during the year of policy implementation is significantly above 0, suggesting an immediate positive effect of the ICPC policy on industrial resilience, with no observed lag. Additionally, examining the trend in the estimated values of $\alpha_{k}$ after the implementation of the ICPC policy reveals a significant divergence in industrial resilience between pilot and non-pilot cities. This divergence illustrates a progressive enhancement in the industrial resilience of pilot cities, which continues to grow year by year.

### 4.3. Placebo test

**Fig 3:**
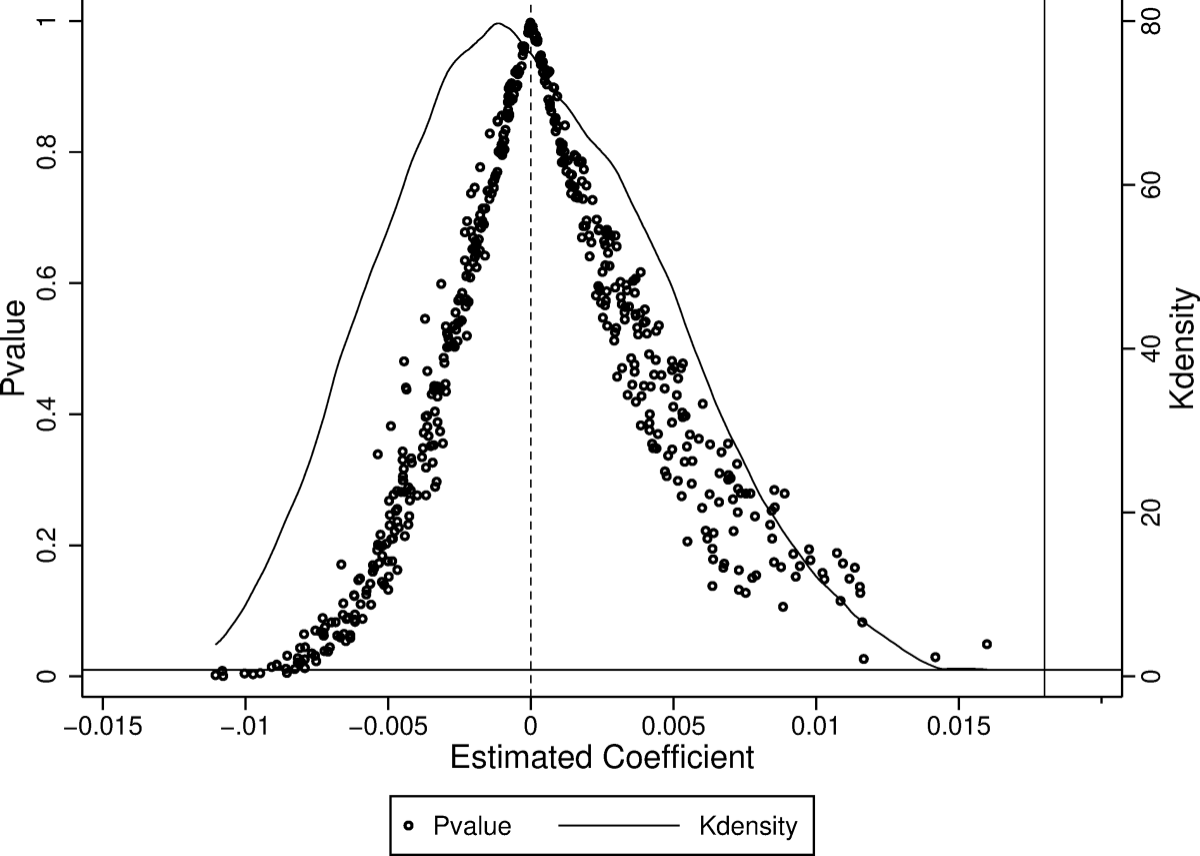
Placebo test.

Despite including control variables, year-fixed, and city-fixed effects in Model (1), its results may still be influenced by unobservable factors. To minimize this impact, we conducted a placebo test using a randomized experimental group approach, following the methodology outlined by Cai et al. [58]. Through this process, we obtained 500 estimates of the coefficients for fictitious information consumption pilot city dummy variables along with their corresponding p-values, as shown in Fig 3. The solid line represents the kernel density distribution of the estimated coefficients for these fictitious variables, while the hollow circles indicate the associated p-values. The solid line parallel to the vertical axis marks the benchmark regression coefficient value, and the dashed line parallel to the horizontal axis represents p = 0.01. The distribution of the estimated coefficients for the fictitious dummy variables exhibits a normal distribution centered around 0. Furthermore, the majority of the corresponding p-values are situated above the dashed line, indicating a lack of statistical significance.

Additionally, there is a clear difference between the distribution of these estimated coefficients and the benchmark regression coefficient estimate (0.018). These results indicate that the placebo test is successful, suggesting that the observed effect of the ICPC policy on enhancing industrial resilience is not due to chance or unobservable factors.

### 4.4. Endogeneity test

The benchmark analysis has established that the ICPC policy significantly enhances urban industrial resilience. While the likelihood of endogeneity arising from reverse causality is relatively low, this study aims to eliminate any potential influence by employing an instrumental variable (IV) approach, following the methodology of Hui and Xujun [24]. Specifically, we utilize the interaction term between the number of fixed-line telephones per 100 people in 1984 and a time dummy variable as our instrumental variable. On one hand, information consumption relies on digital devices and mobile internet as primary carriers. Therefore, the historical number of fixed-line telephones per 100 people has, to some extent, established a foundation for regional digital consumption in terms of equipment and supporting infrastructure, thus fulfilling the relevance requirement of the *IV*. On the other hand, as fixed-line telephones have been increasingly replaced by modern technologies, they are unlikely to directly affect urban industrial resilience in the current digital landscape, thereby satisfying the homogeneity requirement. Table 3 presents the regression results of the 2SLS method. Column (1) shows that the *IV* is valid. Additionally, both the coefficients of IV and Did_inf are significantly positive at the 1% level. This finding indicates that, after addressing potential endogeneity issues, the positive impact of the ICPC policy on urban industrial resilience remains robust, thereby confirming that Hypothesis H1 is upheld.

**Table 3:** Endogeneity test.

	Phase I	Phase II
	Did_inf	**Res**
IV	0.008^***^	
	(4.108)	
Did_inf		0.638^***^
		(4.081)
Cragg-Donald F	18.410^***^	
Kleibergen-Paap rk LM	16.875^***^	
Controls	YES	YES
City-FE	YES	YES
Year-FE	YES	YES
N	2592	2592

### 4.5 Robustness tests

#### 4.5.1 Excluding the effects of other policies.

To address the potential impact of concurrent policies during the sample period on the baseline results, this study adopts the approach of Zhang et al. [30] by carefully reviewing the policy objectives in place during the study timeframe. We specifically exclude interference from three policies that are closely related to information infrastructure development: “Broadband China,” “National E-commerce Demonstration Cities,” and “Big Data Comprehensive Pilot Zones.” Table 4 presents the estimated effects of the ICPC policy on industrial resilience after incorporating each of these three interfering policies in Columns (1) to (3). The results show that after controlling for the influence of other information policies, the coefficients of the Did_inf remain positive and significant at the 1% level. This indicates that the ICPC policy continues to effectively enhance industrial resilience, even when accounting for the effects of concurrent policies.

**Table 4:** Excluding other policy effects.

	(1)	(2)	(3)
	**Res**	**Res**	**Res**
Did_inf	0.015***	0.013***	0.019***
	(3.010)	(3.063)	(3.693)
Broadband	0.012***		
	(3.018)		
E−commerce		0.036***	
		(6.208)	
Bigdata			0.017**
			(2.136)
Controls	YES	YES	YES
City-FE	YES	YES	YES
Year-FE	YES	YES	YES
N	3372	3372	3372
R^2^	0.754	0.769	0.755

#### 4.5.2. Heterogeneity treatment effect test.

Since the pilot cities were not established simultaneously but constructed in phases, the estimation results of the baseline model may be subject to TEFW fixed effects bias. To address this issue, this study employs two key approaches: Bacon decomposition and heterogeneity-robust estimators. First, the Bacon decomposition method proposed by Goodman-Bacon [59] is used to examine potential heterogeneity in treatment effects. The results, presented in Panel A of Table 5, show that the weight of the inappropriate treatment effect (Later T vs. Earlier C) is only 6.3%. This indicates that the heterogeneity of treatment effects in the overlapping DID model is minimal, suggesting that the benchmark regression results are robust.

**Table 5:** Heterogeneity treatment effect results.

Panel A Bacon decomposition results		
**Grouping**	**Weight**	**Coefficient**
Earlier T vs. Later C	0.050	0.015
Later T vs. Earlier C	0.063	-0.024
T vs. Never treated	0.887	0.023
**Panel B Heterogeneity-robust estimators**		
**Approach**	**Did_inf**	
De Chaisemartin and D’Haultfoeuille. (2020)	0.014^***^ (3.852)	
Sun and Abraham (2021)	0.026^***^ (3.631)	
Borusyak et al. (2024)	0.026^***^ (3.807)	

Second, to further mitigate the potential impact of heterogeneous treatment effects on the findings, three state-of-the-art robust estimators from recent research are applied. These include: (1) The method proposed by De Chaisemartin and D’Haultfoeuille [60], which addresses the bias caused by staggered treatment adoption and provides unbiased estimates under complex time distributions. (2) The interaction-weighted estimator developed by Sun and Abraham [61], which accounts for heterogeneous treatment effects across groups and time periods. (3) The interpolation-based counterfactual method introduced by Borusyak et al. [62], which corrects for TWFE estimation bias and ensures robustness in multi-period settings. As shown in Panel B of Table 5, the regression coefficients of *Did_inf* are all significant at the 1% level across all three methods. These results confirm that information consumption has a significant positive effect on industrial resilience, even after accounting for potential biases caused by heterogeneous treatment effects. In conclusion, by addressing TEFW bias and heterogeneous treatment effects through Bacon decomposition and multiple robust estimators, strong evidence is provided to support the positive impact of information consumption on industrial resilience.

#### 4.5.3. Other robustness tests.

To further validate the robustness of the baseline conclusions, we conduct a series of robustness checks from several perspectives:

(1) Addressing selection bias. Policymakers might have prioritized cities with advanced economic development and information infrastructure when selecting information consumption pilot cities, leading to inherent differences between pilot and non-pilot cities. To address this, we use the control variables in Model (1) as covariates and employ caliper matching and nearest neighbor matching methods to match the treatment group annually. The results of the PSM-DID analysis are presented in Columns (1) and (2) of Table 6.
(2) Winsorization. To avoid potential biases caused by extreme values of the explained variable, this study applies a 1% winsorization treatment to both the upper and lower tails of the distribution and re-estimates the model using the trimmed sample. The results are shown in Column (3) of Table 6.
(3) Exclusion of special cities. Recognizing the significant differences in economic development and resource allocation between municipalities such as Beijing, Shanghai, and other cities, this study excludes these four municipalities from the sample and re-runs the regression. The results are presented in Column (4) of Table 6.
(4) Stringent clustering. To further mitigate the potential impact of heterogeneity among cities on the results, this study adjusts the clustering method to a stricter provincial level and re-estimates the model. The results are displayed in Column (5) of Table 6.
(5) Controlling for provincial-year interaction effects. City-level data are often influenced by various provincial policies that vary over time. These provincial-level factors, if uncontrolled, may act as omitted variables and bias the estimation results. Therefore, we control for the interaction terms between provinces and years in the baseline model to reduce errors caused by omitted variables. The results are reported in Column (6) of Table 6.

**Table 6:** Other robustness tests.

	(1)	(2)	(3)	(4)	(5)	(6)
	**Res**	**Res**	**Res**	**Res**	**Res**	**Res**
Did_inf	0.009^**^	0.009^**^	0.014^***^	0.013^***^	0.018^***^	0.014^***^
	(1.982)	(2.053)	(3.580)	(3.042)	(3.872)	(3.257)
Controls	YES	YES	YES	YES	YES	YES
City-FE	YES	YES	YES	YES	YES	YES
Year-FE	YES	YES	YES	YES	YES	YES
Prov*Year	NO	NO	NO	NO	NO	YES
N	2686	2741	3372	3324	3372	3372
R^2^	0.738	0.739	0.810	0.739	0.752	0.771

The results consistently show that, regardless of the method used to address sample selection bias, mitigate the influence of extreme values, or control for omitted variables, the coefficient for Did_inf remains positive and significant at least at the 5% level. This indicates that the positive effect of the ICPC policy on enhancing industrial resilience holds and is robust across a variety of robustness checks.

## 5. Further analysis

### 5.1. Mechanism tests

The previous empirical results indicate that the ICPC policy significantly enhances urban industrial resilience. To further establish causality, this section conducts a mechanism analysis. Based on theoretical insights, information development, entrepreneurial agglomeration, and digital finance serve as critical channels through which the ICPC policy influences industrial resilience. Therefore, this section explores these three mechanisms in order to examine the pathways through which ICPC policies enhance industrial resilience.

(1) Information development effect. According to theoretical analysis, the ICPC policy can promote urban informatization by enhancing digital infrastructure and fostering innovation, which in turn strengthens industrial resilience. If this mechanism holds, the impact of the ICPC policy on industrial resilience should be more pronounced in cities with greater informatization development. To verify this, we use the proportion of employees in the information transmission, computer services, and software industries relative to total employment (*Inter*) as an indicator of informatization. Cities are classified into high and low informatization groups based on whether their average informatization level over the sample period. The results in Table 7 columns (1) and (2) show that in cities with a low level of informatization, the coefficient of *Did_inf* is 0.009. In cities with a high level of informatization, the coefficient increases to 0.032 and is significant at the 1% level. These findings suggest that the ICPC policy has a stronger effect on industrial resilience in cities with higher information levels, supporting Hypothesis H2.
(2) Entrepreneurial agglomeration effect. Theoretically, the ICPC policy fosters a better entrepreneurial environment by improving business conditions, financial support, and innovation incentives, thereby strengthening industrial resilience. If this mechanism holds, the impact of the ICPC policy should be more pronounced in cities with higher levels of entrepreneurial agglomeration. To test this, following the approach of Hui and Xujun. (2024) [24], we use the number of newly established enterprises per 100 people as an indicator of entrepreneurial agglomeration (*EA*). Following the same classification method, cities are grouped into high and low entrepreneurial agglomeration categories. The results in Table 7 columns (3) and (4) indicate that in cities with low entrepreneurial agglomeration, the coefficient of *Did_inf* is not statistically significant. However, in cities with high entrepreneurial agglomeration, the coefficient of *Did_inf* is 0.018 and significant at the 1% level. This implies that the ICPC policy significantly enhances industrial resilience in cities with high levels of entrepreneurial agglomeration, verifying Hypothesis H3.
(3) Digital finance effect. From a theoretical perspective, the ICPC policy can promote digital finance (*DF*) by expanding financial accessibility, fostering digital payment systems, and supporting fintech innovations, which in turn bolster industrial resilience. If this mechanism is valid, the ICPC policy should have a more significant effect on industrial resilience in cities with higher levels of digital finance development. To measure this, we use the Digital Inclusive Finance Index published by Peking University. Following the same classification method, cities are grouped into high and low digital finance categories. As shown in Table 7 columns (5) and (6), in cities with low digital finance development, the coefficient of *Did_inf* is 0.002 and not statistically significant. In contrast, in cities with high digital finance development, the coefficient of *Did_inf* is 0.018 and significant at the 5% level. These results indicate that the ICPC policy strengthens industrial resilience more effectively in cities with a well-developed digital finance infrastructure, confirming Hypothesis H4.

**Table 7:** Mechanism test results.

	(1)	(2)	(3)	(4)	(5)	(6)
	**Inter-Low**	**Inter-High**	**EA-Low**	**EA-High**	**DF-Low**	**DF-High**
Did_inf	0.009^*^	0.032^***^	0.002	0.018^***^	0.002	0.018^**^
	(1.878)	(2.983)	(1.319)	(2.605)	(1.205)	(2.147)
Controls	YES	YES	YES	YES	YES	YES
City-FE	YES	YES	YES	YES	YES	YES
Year-FE	YES	YES	YES	YES	YES	YES
N	2328	1044	1464	1908	1956	1416
R^2^	0.694	0.785	0.289	0.740	0.234	0.765

### 5.2. Heterogeneity test

#### 5.2.1. Locational heterogeneity.

There are notable regional disparities in economic development, industrial structure, and the degree of information technology adoption, which may result in uneven effects of the ICPC policy across different areas [31]. Columns (1) to (4) of Table 8 display the regression results for the eastern, central, western regions, and central-western respectively. The findings reveal that the policy has no significant impact on industrial resilience in the western regions, while it significantly enhances the resilience of urban industrial chains in both the eastern and central regions. Importantly, the regression coefficient for the eastern cities is the largest and statistically significant at the 1% level. To further examine whether the policy effects differ significantly across regions, we conducted coefficient difference tests between groups. The results indicate that the policy effect in eastern regions is significantly higher than that in central and western regions at the 1% level, while the difference between central-western regions is also statistically significant. These findings highlight the existence of locational differences in policy effectiveness across regions.

**Table 8:** Regional location heterogeneity test results.

	(1)	(2)	(3)	(4)
	**Eastern**	**Central**	**Western**	**Central-Western**
Did_inf	0.035^***^	0.010^**^	0.006	0.007^**^
	(3.509)	(2.346)	(1.429)	(2.501)
Test for differences between groups				
Eastern vs. Central	0.025^***^			
Eastern vs. Western	0.029^***^			
Central vs. Western		0.004^**^		
Eastern vs. Central- Western	0.028^***^			
Controls	YES	YES	YES	YES
City-FE	YES	YES	YES	YES
Year-FE	YES	YES	YES	YES
N	1428	960	984	1944
R^2^	0.773	0.697	0.629	0.651

Possible explanations for this include the stronger economic foundations, optimized industrial structures, and advanced information technology development in eastern regions, which allow them to better leverage the opportunities presented by the ICPC policy. In these cities, information consumption drives the widespread adoption of information technology, facilitating the digital transformation of industrial chains and enhancing innovation capacity [7,21]. In contrast, the central and western regions face challenges such as lower levels of economic development, more monolithic industrial structures, and weaker information technology infrastructure, limiting the effectiveness of the pilot policies due to insufficient external support. The relatively underdeveloped information infrastructure in these regions hinders the penetration and application of information technology in industries [54]. Enterprises in these areas often lack the necessary technical support and financial investment for their digital transformation, leading to limited improvements in industrial resilience through information consumption. As a result, the positive effects of the ICPC policy on industrial resilience are not significantly observed in these cities.

#### 5.2.2. Urban scale heterogeneity.

Large-scale cities and small-to-medium-sized cities differ significantly in terms of economic foundations, market demands, and levels of informatization, which may result in varying outcomes of the ICPC policy in enhancing industrial resilience. To account for these differences, we conduct heterogeneity tests to assess the specific policy impacts across different city sizes, ensuring the precision and effectiveness of policy implementation. Following the classification method of Liu et al. (2024) [5], cities are categorized into large-scale and small-sized cities based on their permanent resident populations. Columns (1) and (2) of Table 9 present the regression results for the impact of the ICPC policy on industrial resilience in large and small cities, respectively. The results indicate that the regression coefficients of the ICPC policy (*Did_inf*) are significantly positive for both large and small cities, though the coefficient for large cities is notably higher. Specifically, the estimated coefficient for large cities is 0.025, significant at the 1% level, while for small cities, the coefficient is 0.008, significant at the 10% level. This suggests that the ICPC policy has a more pronounced effect on enhancing industrial resilience in large cities. Moreover, the coefficient difference test confirms that the impact of the ICPC policy is significantly greater in large cities than in small cities at the 1% significance level.

**Table 9:** City size heterogeneity test results.

	(1)	(2)
	**Large**	**Small**
Did_inf	0.025^***^	0.008^*^
	(3.072)	(1.795)
Test for differences between groups	0.017^***^	
Controls	YES	YES
City-FE	YES	YES
Year-FE	YES	YES
N	1399	1973
R^2^	0.808	0.670

Possible reasons for this include the more advanced infrastructure, richer industrial resources, and stronger innovation capacities typically found in large cities, which may amplify the positive impact of the ICPC policy [5,37]. Large cities generally have better access to financial resources, a higher concentration of technology-intensive industries, and a more dynamic business environment, all of which contribute to a stronger policy response. In contrast, small cities may face constraints such as weaker industrial foundations, lower levels of digitalization, and limited access to high-quality human capital, which could hinder the full realization of policy benefits. The significant disparity in policy effects across city sizes underscores the importance of tailored policy strategies that consider the unique characteristics and development needs of different urban areas.

## 6. Discussion

Prior studies have predominantly examined the impact of information consumption on economic growth and urban green development, emphasizing its positive role in fostering a green economy [5,30]. In contrast, this study focuses on urban industrial resilience, adopting a demand-side perspective to uncover the positive effects of new forms of consumption on regional economic development in the digital economy era. As a key component of economic and overall urban resilience, industrial resilience reflects a city’s capacity to withstand and recover from external shocks, encompassing both resistance and recovery capabilities [46]. Enhancing industrial resilience ensures stable long-term economic growth, even in the face of economic crises and uncertainties, aligning well with the principles of sustainable development in uncertain global landscape. By examining the positive effects of information consumption on urban industrial resilience, this study offers valuable insights and empirical support for policymakers.

From a theoretical perspective, this study incorporates both resistance and recovery dimensions into the measurement of industrial resilience, thereby enriching the methodology used to assess industrial resilience. Prior studies have primarily emphasized supply-side factors—such as technological innovation [37,50], financial development [63], and industrial policies [64]—as key drivers of resilience, while the role of demand-side factors has been largely overlooked. In contrast, this study highlights how digital consumption, driven by information technology, can significantly enhance urban industrial resilience. This demand-side approach broadens the literature by shifting the focus from production-oriented factors to consumption-driven mechanisms, particularly in the context of the digital economy. Additionally, while previous research has recognized the importance of digitalization for economic resilience, few studies have empirically examined the specific channels through which information consumption influences industrial dynamics. By leveraging a quasi-natural experiment, this study fills this gap and provides robust empirical evidence on the relationship between digital consumption and industrial resilience.

From a practical standpoint, the findings of this study provide critical insights for policymakers, not only in China but also in other countries and regions. For China, the primary practical significance lies in how the central government can strategically leverage digital consumption to drive new forms of consumer demand, as well as how local governments can adopt region-specific economic strategies to enhance urban industrial resilience. More detailed policy recommendations will be provided in the following section. What valuable insights does this China-based pilot study offer for other countries and regions? Drawing on the literature review and the diversity of national contexts, this study presents the following international perspectives. First, it confirms the positive impact of information consumption on industrial supply chains. Based on this, governments worldwide should actively engage in the research, development, and promotion of digital technologies, particularly by strengthening digital infrastructure and fostering an environment conducive to technological application. By driving new consumer demand, countries can effectively ensure the security and stability of urban industrial supply chains, while international cooperation can contribute to strengthening the resilience of the global industrial system. Second, governments should formulate and implement policies tailored to their respective levels of digital technology development and industrial growth. During policy implementation, policymakers should leverage regulatory and optimization mechanisms to continuously adjust and refine policies based on practical outcomes. This iterative approach will help maximize the positive impact of digital consumption on industrial resilience and ensure its long-term effectiveness. Finally, from an economic development perspective, industrial resilience plays a pivotal role in building resilient cities and securing a stable position in global industrial supply chains. Against the backdrop of global deindustrialization, many countries face significant challenges in maintaining industrial productivity. To address these issues, governments should actively enhance the adaptability and recovery capacity of urban industries, thereby fostering sustainable industrial development. Strengthening industrial resilience will lay a solid foundation for the construction of cities that are more capable of withstanding economic shocks and environmental challenges.

## 7. Conclusions and policy implications

### 7.1 Findings

Examining the impact of new forms of consumption, such as information consumption, on urban industrial resilience from the demand side holds significant practical implications for ensuring industrial security and promoting sustainable urban economic growth. Leveraging the quasi-natural experiment of the ICPC policy, this study empirically investigates its impact on urban industrial resilience, its underlying mechanisms, and its heterogeneity using a difference-in-differences (DID) model after confirming the policy’s effectiveness. The key findings are as follows: (1) The ICPC policy significantly enhances urban industrial resilience. This effect remains robust after addressing potential endogeneity, selectivity bias, placebo tests, and accounting for the influence of other policies in a series of robustness checks. (2) The ICPC policy improves urban industrial resilience primarily through mechanisms such as information development, entrepreneurial agglomeration, and the growth of digital finance. (3) The impact of the ICPC policy exhibits heterogeneity, with more pronounced effects observed in the eastern and central regions, as well as in larger cities. This study not only extends the theoretical perspective of demand-side influences on industrial resilience, but also provides sufficient empirical evidence and insights for enhancing industrial resilience through rigorous empirical analysis.

### 7.2. Policy implications and recommendations

To further strengthen the positive impact of the ICPC policy on urban industrial resilience and promote the broader expansion of its effects, this paper proposes the following policy recommendations based on the research findings: Firstly, it is essential to continue promoting and deepening the ICPC pilot initiatives to enhance the digitalization of consumption. The benchmark findings of this study indicate that the ICPC policy has significantly contributed to improving urban industrial resilience. Therefore, it is critical to further advance information consumption. Policymakers should actively encourage and support innovations in digital consumption models within pilot cities to attract a greater number of businesses and consumers through diversified digital consumption scenarios. Concurrently, investment in digital infrastructure should be increased to facilitate the widespread adoption and optimization of digital consumption platforms. This approach will enable the deep integration of information technology with consumption scenarios, thereby fostering the continued optimization of urban digital applications and the upgrading and transformation of residents’ consumption patterns.

Secondly, policymakers and government departments should prioritize the agglomeration of talent and entrepreneurial resources, optimize resource allocation, and foster deep cooperation between industry and finance. Local governments need to formulate and enhance relevant policies that focus on cultivating digital talent, encouraging enterprises to increase their investments in information technology, and promoting the widespread application of information technology across various industries to stimulate technological innovation. Additionally, it is crucial to strengthen support for the development of industrial clusters by optimizing resource allocation and fostering technological cooperation within regions, thereby achieving higher-quality industrial resilience. Furthermore, the government should actively guide and support the widespread adoption of financial technology in small and medium-sized enterprises, encouraging these firms to undergo digital transformation to enhance their market competitiveness and resilience to risks.

Additionally, local governments should enhance the support conditions for the ICPC policy by taking into account the specific characteristics of their regions. Currently, the uneven development of information infrastructure across various regions in China hampers the effective flow of production factors, while information asymmetry and market entry barriers obstruct the effective supply of digital consumption. Against this complex backdrop, cities should look to their own circumstances to maximize the industrial resilience-enhancing effects of information-consuming cities. Specifically, in regions and cities where the economic and technological conditions are favorable—such as in the eastern regions and large cities—policymakers should focus on further integrating digital consumption with high-end manufacturing and service sectors, promoting advanced technological applications, and ensuring that the benefits of digitalization are fully harnessed. In addition, the government should encourage enterprises to increase their investment in corporate R&D, promote collaborative innovation among enterprises upstream and downstream of the industrial chain, and collaboratively promote the improvement of industrial resilience. Conversely, in regions with less developed infrastructures, particularly in western areas and smaller cities, priority should be given to enhancing digital infrastructure and building local capacity for technology adoption. Such measures could include targeted investments in digital networks, support for local technology ventures, and programs aimed at attracting and retaining skilled digital talent. To solve the problem of insufficient external support, a linkage mechanism between the government, enterprises and financial institutions should be established, and special support funds for technological progress should be set up to guide the clustering of resources and provide a favorable external environment for the stability of the industrial chain. In addition, these regions should establish a cross-regional collaborative innovation platform to attract the transfer of experience and technology from cities in the eastern region to the central and western regions, share resources and narrow the regional development gap. In addition, the formulation and implementation process of policy implementation should play a regulatory and optimization effect, tailored to the characteristics of different regions to ensure the long-term effectiveness of the policy industrial resilience enhancement effect, thereby promoting urban resilience.

### 7.3. Limitations and shortcomings

While this study offers valuable insights into the positive impact of the ICPC policy, certain limitations remain. The economic promotion effects observed in pilot cities may not be fully generalizable to all urban types, given the diverse regional characteristics. Moreover, although using information consumption as a proxy for new consumption is a reasonable approach, it may not completely capture the intensity and structural shifts in the digital economy. Furthermore, regarding the measurement of industrial resilience, there is no consensus in the academic community, and future research should further strengthen the measurement of this variable. Future research should consider incorporating a broader range of policy indicators and more diverse regional samples to provide a more comprehensive analysis of information consumption and its influence on urban industrial resilience.
